# Potential of Rule-Based Methods and Deep Learning Architectures for ECG Diagnostics

**DOI:** 10.3390/diagnostics11091678

**Published:** 2021-09-14

**Authors:** Giovanni Bortolan, Ivaylo Christov, Iana Simova

**Affiliations:** 1 Institute of Neuroscience IN-CNR, Corso Stati Uniti 4, 35127 Padova, Italy; giovanni.bortolan@cnr.it; 2 Institute of Biophysics and Biomedical Engineering, Bulgarian Academy of Sciences, Acad. G. Bonchev Str. Bl 105, 1113 Sofia, Bulgaria; 3 Heart and Brain Center of Excellence, University Hospital Pleven, Pierre Curie 2 Str, 5804 Pleven, Bulgaria; ianasimova@gmail.com

**Keywords:** ECG, arrhythmia, features, rule-based method, convolutional neural network, GoogLeNet network, wavelet transform, scalogram, PhysioNet/Computing in Cardiology Challenge 2020

## Abstract

The main objective of this study is to propose relatively simple techniques for the automatic diagnosis of electrocardiogram (ECG) signals based on a classical rule-based method and a convolutional deep learning architecture. The validation task was performed in the framework of the PhysioNet/Computing in Cardiology Challenge 2020, where seven databases consisting of 66,361 recordings with 12-lead ECGs were considered for training, validation and test sets. A total of 24 different diagnostic classes are considered in the entire training set. The rule-based method uses morphological and time-frequency ECG descriptors that are defined for each diagnostic label. These rules are extracted from the knowledge base of a cardiologist or from a textbook, with no direct learning procedure in the first phase, whereas a refinement was tested in the second phase. The deep learning method considers both raw ECG and median beat signals. These data are processed via continuous wavelet transform analysis, obtaining a time-frequency domain representation, with the generation of specific images (ECG scalograms). These images are then used for the training of a convolutional neural network based on GoogLeNet topology for ECG diagnostic classification. Cross-validation evaluation was performed for testing purposes. A total of 217 teams submitted 1395 algorithms during the Challenge. The diagnostic accuracy of our algorithm produced a challenge validation score of 0.325 (CPU time = 35 min) for the rule-based method, and a 0.426 (CPU time = 1664 min) for the deep learning method, which resulted in our team attaining 12th place in the competition.

## 1. Introduction

The automatic detection and classification of cardiac abnormalities from 12-lead ECG signals has been an area of research interest for a long time [1]. Methods have ranged from medical decision-support systems to statistical approaches, from simple neural network architectures to more sophisticated methods based on deep neural networks [1,2,3]. There has been much focus on research employing the use of deep learning with medical images [4], time series classification [5], and object detection [6]. In [7], a deep recurrent neural network approach was developed and tested for the classification of four types of the severity of atrial fibrillation (AF) based on 21 features. The use of continuous wavelet transforms (CWTs) for ECG signal processing is present in several studies; for example, in [8] the CWT was considered for multiscale parameter estimation for delineation of the fiducial points of P-QRS-T waves.

Recent examples of diagnostic 12-lead ECG classification have been reported. They come from the use of a deep neural network for the classification of six diagnostic classes [3], whereas the study in [9] considered the analysis of 12-lead ECG signals based on deep learning for the classification of four types of arrhythmias. A deep learning neural network model was tested in a database of 6788 12-lead ECG records for the identification of nine diagnostic classes [10].

Consequently, many algorithms may be used to identify cardiac abnormalities. However, most of these methods are trained, tested or developed in relatively small or homogeneous databases, and most of them focus on identifying a small number of cardiac arrhythmias that do not represent the full complexity of ECG classifications [11]. After a long series of interesting annual challenges, the PhysioNet/Computing in Cardiology Challenge 2020 provided the opportunity to address these problems, considering an extended set of diagnostic classes and a set of learning/testing ECG records belonging to different databases [11,12,13].

The main objective of this study was to test two different techniques for the automatic classification of ECG signals with active participation in the PhysioNet/Computing in Cardiology Challenge 2020. In particular, the classical rule-based system method, as well as a more sophisticated technique based on direct learning from ECG raw data through deep learning architectures, are explored and compared in the same framework.

## 2. Materials and Methods

### 2.1. ECG Database

The PhysioNet/Computing in Cardiology Challenge 2020 provided a training set of six databases (Table 1) with 43,101 annotated recordings of 12-lead ECGs, lasting from 6 to 60 s [11,14,15,16,17]. In addition, the Challenge involved a seventh undisclosed dataset from an American institution that was geographically distinct from the other datasets, which was used as a test set. Then, a total of 23,260 ECG records, kept hidden, were used for validation (6630) and test (16,630) procedures. In Table 1, the heterogeneity of the databases is evident, considering different sets of diagnostic classes.

The initial 111 diagnoses or classes were further reduced to the 27 diagnostic classes considered in the Challenge scoring system (see Table 2 for a full list of the diagnoses and codes). They are reduced to 24 when considering three equivalent classes.

The composition and the number of annotated diagnostic classes of the six considered datasets for the learning phase are reported in Table 1. The collection of this large training dataset consists of a total of 43,101 ECG recordings and 60,373 diagnostic instances. This means that in every record of the entire database, there is a mean of 1.4 diagnostic classes.

Table 2 shows the distribution of the 24 different diagnostic classes considered in the entire learning set. As can be seen in this table, the number of training instances of the various diagnostic classes is not uniform, with the evident presence of a class imbalance. For example, the NSR class is present in 20,846 records, whereas the Bradycardia class consists of only 288 instances. In addition, there are seven (29.1%) diagnostic classes (AFL, Brady, PR, LPR and RAD, LQRSV, PVC) with a number of records lower than 600 and 16 classes (66.6%) with a number of records higher than 1000. For this reason, a strategy of under-sampling for a more uniform distribution of the classes was adopted. For the selection of representative learning subsets, a random selection of ECG records with at most N_max instances for the considered classes was determined. Three values of N_max were considered and tested: 600, 1000 and 1500, obtaining the learning subsets LS_N600, LS_N1000 and LS_N1500, as described in Table 3. The weighted distribution of the learning subset LS_N1000, consisting of 16,002 ECG records, is reported in the rightmost column of Table 2, which shows a slightly more equilibrated distribution for the learning phase. All ECG data were resampled at 500 Hz (if necessary) for compatibility purposes.

### 2.2. Preprocessing

The ECG recordings were filtered to suppress the power-line interference, the drift of the isoelectric line and the electromyographic noise. QRS detection was performed via the identification of significant peaks of spatial velocity (absolute value of the first derivative of one or more leads), using combined thresholds, with the subsequent identification of the R waves and the computation of the heart rate [18]. Then, a robust average beat is calculated, with reference to the positive (R), or negative (S) peaks with the highest amplitude, through the signal-averaging of the sustained beats in the record. The rejected outliers are suspected to be artefacts or abnormal beats with non-sustained amplitudes.

### 2.3. Rule-Based Classifier

Manual interpretation of the electrocardiogram is time-consuming and requires skilled personnel with a high degree of training [11]. Although the knowledge of an expert is complex to formalize, we tried to develop a rule-based method to mimic some simple rules. This method uses morphological and time-frequency ECG descriptors, characterizing each diagnostic label. These rules have been extracted from the knowledge-base of a physician or from a textbook [19], with no direct learning procedure in the first phase, although a refinement was tested in the second phase.

After QRS detection and computation of the median of all beats, the next step considered the delineation of QRS-onsets and QRS-offsets, and the identification of T_end and heartbeat classification [20,21]. In addition, some parameters were computed in the derived vectorcardiographic (VCG) signal [22,23].

**Figure 1:**
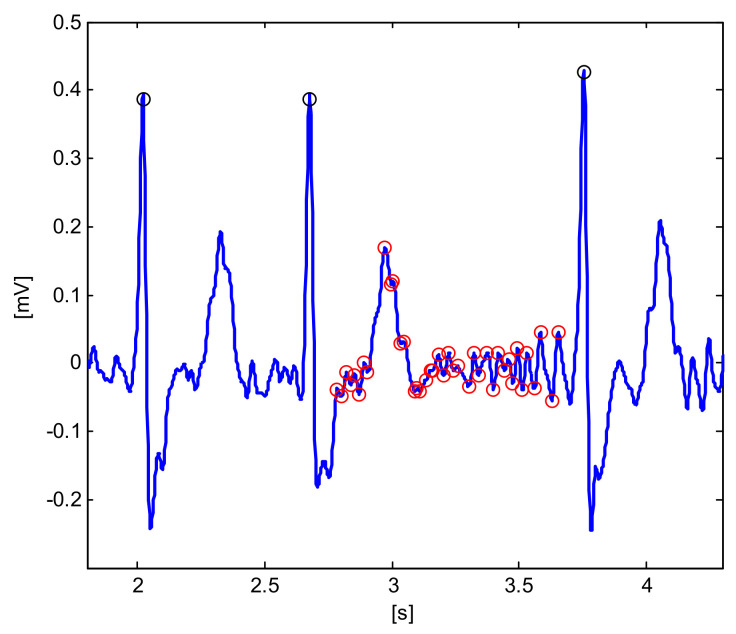
Signal processing for the detection of AF/AFL (file A02430, AF annotation, Lead II). Markers denote: detected QRS (o-black), zero-line crossing of the first derivative (o-red).

**Figure 2:**
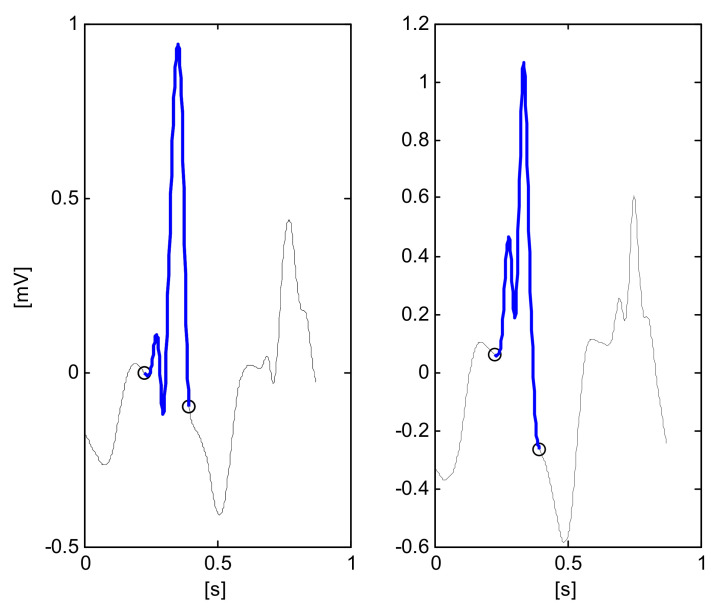
Signal processing for the detection of RBBB (file H03947, RBBB annotation, leads V2-left and V3-right). Markers denote: QRSon and QRSoff (o-black), QRS segmentation that resembles an ‘M’ shaped QRS (blue trace).

The main parameters, computed using classical algorithms, are reported in Table 4. Figure 1 reports some components of the signal processing for the detection AF/AFL, with the identification of zero-line crossing of the first derivative. Figure 2 reports two examples of QRS segmentation that resemble an ‘M’ shaped QRS, which is a particular step for the detection of RBBB.

From these parameters, the considered diagnostic rules are represented and described in the following “**if**–**then**” format:
**PAC****if***std_RR* in *[35 ms–46 ms]*
**then**
*PAC = 0.7*
**if***std_RR > 0.46 ms***then***PAC = 1***PVC****if**max_QRS_ampl > 2 * mean_QRS_ampl**then***PVC = 1*
**if**min_QRS_ampl < 0.5 * mean_QRS_ampl**then***VC = 1***STach****if***PVC = 0 and PAC =0 and mean_RR< 600 ms***then***STach = 1***Brady****if***PVC = 0 and PAC = 0 and mean_RR > 1000 ms***then***Brady = 1***SA****if***PAC = 1 and PVC = 0***then***SA = 1***SNR****if***PAC = 0 & PVC = 0***then***SNR = 1***SB****if***Brady = 1 and SNR = 1***then***SB = 1***AFL****if***PAC > 0.7 and n_cross_outside_QRS > 9***then***AFL = 1 and AF = 0***AF****if***PAC > 0.7 and n_cross_outside_QRS > 15***then***AF = 1 and AFL = 0***LBBB****if***QRS_dur > 120 ms and no_Q_wave in I, V6 and pred_R_wave in I, V6, and opp_ST_T in V5***then***LBBB = 1***RBBB****if***QRS_dur > 100 ms and MW_shaped_QRS in any of V1,V2,V3,V4 and n_cross_inside_QRS ≥ 5***then***RBBB = 1***IRBBB****if***RBBB = 1 and QRS_dur < 120 ms***then***IRBBB = 1***RAD****if***QRS_angle is in the range [90°,180°]***then***RAD = 1***LAD****if***QRS_angle in the range [270°,330°]***then***LAD = 1***LAnFB****if***RAD = 1 or LAD = 1 and (ratio_Q_R < 1/9 or ratio_R_S < 1/9*) **then***LAnFB = 1***PR****if** no*_P_wave and (RAD = 1 or LAD = 1) and slope_ini_QRS > 0.7 mV/ms*
**then**
*PR = 1***LQT****if***QT_int > 340 ms***then***LQT = 1***LQRSV****if***max_QRS_ampl in I, II, III < 0.25 mV***then***LQRSV = 1***I_AVB****if***yes_P_wave and PR_interval > 130 ms***then***I_AVB = 1***LPR****if***yes_P_wave and PR_interval > 110 ms***then***LPR = 1***Tinv****if***T_neg in V1 and T_neg in V2 and T_neg in V3***then***Tinv = 1*

### 2.4. Deep Learning Network Classifier

The deep learning method considers both raw ECG signals and previously computed median beat signals. It is composed of continuous wavelet transforms (CWTs), followed by a convolutional neural network (CNN). The input data of the CWTs are the concatenation of two components of raw ECG signals:

- Concatenation of 10 s of the ECG signals of eight independent leads;
- Concatenation of average beats computed previously using the rule-based method.

These data are processed by the CWTs, obtaining a time-frequency domain representation, with the generation of specific 2D images. These images are then used for the training of a CNN network for ECG diagnostic classification. A pretrained image CNN classification network that has already learned to extract powerful and informative features from natural images has been used as a starting point to train the specific classifier for 24 classes [24].

**Figure 3:**
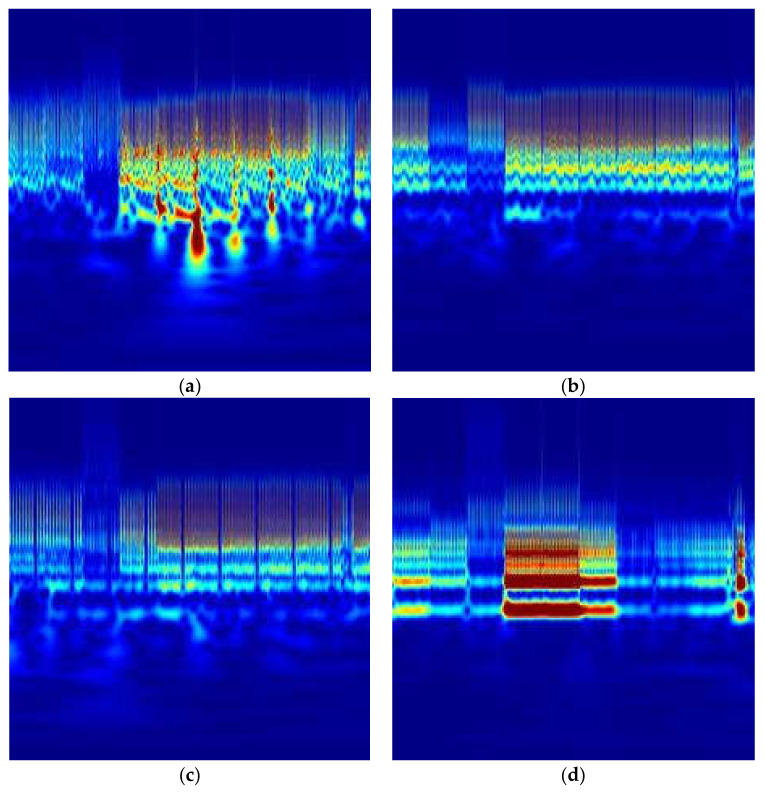
Example of ECG scalograms: (**a**) A0442—Atrial Fibrillation, (**b**) A0453—Normal Sinus Rhythm, (**c**) A0764—Left Bundle Branch Block, (**d**) A0627—Premature Atrial Contraction.

The CWT transforms the selected window of ECG signals into time-frequency representations, which compose a 2D image. In particular, the absolute values of the CWT coefficients of the considered ECG signal have been considered, obtaining the so-called scalogram [24]. The Matlab function cwtfilterbank was used to create the continuous wavelet transform filter bank, using a family of exactly analytic wavelets (Morse wavelet), with symmetry = 3, and time-bandwidth product = 60. In addition, CWT scales are discretized using 12 voices per octave. Two examples of scalograms are shown in Figure 3a (Atrial Fibrillation AF) and in Figure 3b (Normal Sinus Rhythm NSR), where the x-axis represents the time, the y-axis the frequency and the color map is the magnitude.

These models were pretrained on a subset of the ImageNet database (www.ImageNet.org, accessed on 1 August 2021), which was used in the ImageNet Large-Scale Visual Recognition Challenge (ILSVRC) [24,25]. Both networks, trained on ImageNet, are able to classify images into thousands of object categories, learning rich feature representations for a wide range of images. Thanks to the generalization property common to the neural network approach, it is possible to develop an appropriate learning procedure to force the networks to classify images of a different domain produced by the CWT block into 24 diagnostic classes. Both networks were tested in the first phase of the Challenge: the SqueezeNet showed a faster training procedure, whereas the GoogLeNet presented a better performance, and thus the latter was used in the official phase.

GoogLeNet is a CNN, including 22 layers, pretrained to classify images into 1000 object categories. Each layer can be considered as a filter; consequently, the first ones characterize more common features, whereas the deeper ones characterize more specific features in order to differentiate between the considered diagnostic classes.

The learning procedure is characterized by the initial learning rate = 0.0001, a mini-batch size in the interval [30:50] as the minimum factor of the number of elements in the learning set, a variable number of iterations in the various experiments, and the use of the stochastic gradient descent optimization algorithm with momentum (=0.9).

Specific techniques have been implemented and developed during the training phase of the GoogLeNet CNN in order to cope with the particular aspects of multi-label, multi-class characteristics/features. Among different techniques related to class imbalance [26], in the present study, two data-level methods were used: a random under-sampling for reducing the size of the learning sets, and a random over-sampling for duplicating random samples from the minority group.

**Figure 4:**
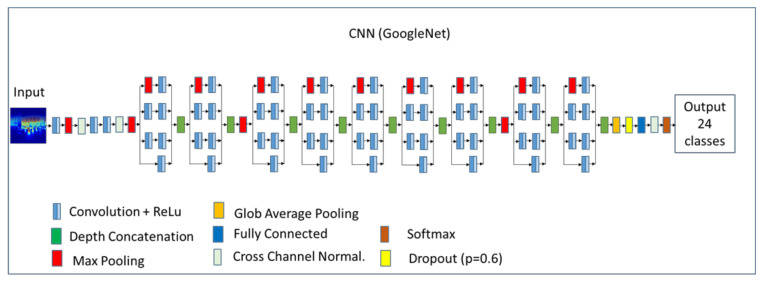
Schematic architecture of the GoogLeNet CNN.

A schematic representation of the architecture of the GoogLeNet network is shown in Figure 4, where the input is a 2D jpeg image (224 × 224 × 3), and the output is represented by the scores or probabilities of the 24 considered ECG classes. An example of a trained CNN GoogLeNet is available at https://github.com/giovanni-ivaylo/cinc20.git (accessed on 1 August 2021) for a complete analysis of its structure.

## 3. Results and Discussion

The score indices of the first and second phase of the Challenge (validation scores) are defined and reported in [11]. In particular, based on the indices of true positive (TP), true negative (TN), false positive (FP), and false negative (FN), precision (TP/(TP + FP)) and recall (TP/(TP + FN)) the following indices were considered:

F_1_ is a F-measure, which is the harmonic mean of precision and recall:
  F_1_ = 2 * TP/(2 * TP + FP + FN)
F_2_, a more general F-measure which weighs recall more highly than precision:
  F_2_ = ((1 + 2^2^) * TP)/((1 + 2^2^) * TP + FP + 2^2^ * FN)
G_2_, a general G-measure:
  G_2_ = TP/(TP + FP + 2 * FN)
AUROC: area under the receiver operating characteristic (ROC) curve
AUPRC: area under the precision-recall curve.
Our team, named ‘Gio_Ivo’, participated successfully in the unofficial and official phases of the Challenge.

In a preliminary phase, the learning process was based only on the CPSC database, consisting of 6877 ECG records with only nine possible diagnostic classes, with a consequent simplification both of the rule-based method and the architecture of the CNN. Table 5 displays the cross-validation indices of the tested algorithms in this preliminary dataset. 

In the official Challenge phase, the entire learning set of 43,101 ECG records was considered, and the number of diagnostic classes increased to 110. The challenge scoring system was essentially concentrated on a subset of 27 classes, considering the relevant diagnostic classes of clinical interest. A particular scoring system was defined by the Challenge for coping with the fact that not all misdiagnosed results are equally bad. In addition, a subset of 24 classes was activated in the identification process, considering three equivalent classes (CRBBB and RBBB, PAC and SVPB and PVC and VEB). During this official phase, the submissions were tested on the validation set of 6630(1463 + 5167) records. To increase the efficiency of the learning process, the learning subsets LS_N1000 (16,002 records), LS_N600 (11,210 records), and LS_N1500 (20,044 records) were used in the testing procedures (Table 3). Table 2 shows the weighted distribution of the learning set LS_N1000 in the 24 diagnostic classes considered.

The deep learning process was performed and tested using three-fold cross-validation techniques. This choice was mainly due to the CPU time required for the training. For example, for a one-fold training iteration, the execution took from 15 to 24 h of CPU time. However, in the submitted algorithms, the presence of several platform-related problems slowed the training process, and consequently, the learning was performed one-fold to ensure an acceptable duration of the learning process and a more convenient feedback phase.

Table 6 reports the official Challenge Validation score of the submitted algorithms tested in the validation set of 6630 records. The rule-based method RB1 essentially did not use any learning process from the database LS_N1000 and the score was in agreement with the behavior of the first phase, whereas the second version (RB2) tried to extract some information from LS_N1000. For example, it tried to differentiate AF from AFL on the basis of the AF-waves’ frequency and amplitude, but the consequent improvement was not significant.

Different deep learning algorithms were submitted, with different learning subsets (LS_N1000, LS_N600, LS_N1500) and a different number of iterations, but the scores (Table 6) were all in the range of [0.400, 0.426], indicating that all these algorithms showed similar behavior. In particular, GoogLeNet_6 resumed the training from a previously saved pretrained network, which comes from a 3-fold cross-validation technique on LS_N1000 and 10 iterations.

Table 7 displays the cross-validation indices trained and tested in the learning databases LS_N1000 and LS_N1500. It is interesting to note that the reported indices F_2, G_2 and the normalized score are in agreement with the official results, with some more optimistic results, probably depending on the composition of the unknown test set.

The final official results were announced considering the test set of 16,630 ECG records. Our team, named ‘Gio_Ivo’, submitted the deep learning method GoogLeNet_6, and achieved a challenge validation score of 0.426 and a full test score of 0.298, thus placing us 12th out of 41 in the official ranking. In particular, Table 8 reports the various official validation score performance indices in the different hidden test/validation sets. The presence of a hidden undisclosed set (10,000 ECG records) from an American institution geographically distinct from the other datasets caused a significant decrease in the Challenge score. This critical point is significant, showing the importance of the composition of the learning/testing sets.

Table 9 shows the AUROC, AUPRC and the F_1_ scores for the considered diagnostic classes. In this table, we can observe the weak points of the classifier. Three diagnostic classes had very low F_1_ scores: Bradycardia (0.0), PR (0.05) and RAD (0.053), which corresponded to the three classes with the lowest numbers of examples (288, 340 and 427, respectively), and also correspondingly low AUPRC values (0.001, 0.019 and 0.025, respectively). These results confirm the critical point of the problem of class imbalance and show the limits of the random over-sampling technique.

The results clearly show that the deep learning architecture that directly examines raw ECG data and time-frequency images is able to produce satisfactory results.

Various teams that participated in the Physionet/Challenge considered the deep learning approach [27,28,29,30], showing a particular interest in this methodology. For example, the team with the highest score [27] considered both raw ECG data and ECG features extracted from ECG signals, including age and gender. A deep neural network with a modified residual neural network architecture was considered in [28], in which the scatter blocks processed the 12 leads separately. In [29], wavelet analysis and a convolutional network were used for each single lead, and a single output label was obtained, reducing the diagnostic categories to the individual and the most frequent combinations. In [30], the authors combined a rule-based model and a squeeze-and-excitation network.

Over recent years, there has been a rapid development of machine learning techniques, with a growing number of ECG classifiers [3,31]. These algorithms consider different sets of cardiac arrhythmias and small or relatively homogeneous datasets, reducing the possibility of a real comparison [11]. For example, in [31] the authors consider 12 classes, in [3] they consider six cardiac abnormalities, whereas the present work considers a set of 24 relevant diagnostic classes of clinical interest, making a direct comparison complex.

Some of the characteristics of the proposed methods can be outlined. The RBM method mimics the classification process of an expert physician, and it obtain the classification in a very short time. However, the accuracy and the mimicking property could be improved with significant effort, considering, for example, some active tuning from the learning database, with more modular rules and fuzzy thresholds. The deep learning method is characterized by the use of a linear architecture fed only with raw ECG data, in which all the leads are examined simultaneously, considering a multi-label classifier with a large number of diagnostic classes, with a positive behavior in the presence of a significant class imbalance. This method has the drawback of complexity and a long training time. The use of pre-trained CNNs has simplified the training process; however, more specific architectures of deep learning could improve the classification accuracy.

## 4. Conclusions

In the present study, we have explored the potential of a classical rule-based method and a deep learning architecture for the automatic classification of ECG signals. The two methods were tested and validated in the framework of the PhysioNet/Computing in Cardiology Challenge 2020, in which six annotated databases of 43,101 ECG records were considered for the training set. The training and validation databases contained a set of 27 relevant diagnostic classes of clinical interest, which represents the complexity and difficulty of ECG interpretation. A particular scoring system was defined by the Challenge judges because not all misdiagnosed classifications are equally bad.

The results of the two different techniques showed that deep learning methods which directly examine raw ECG data and images are able to produce very satisfactory results. In addition, this technique, which is quite a simple methodology but with a high consumption of computation capacity, performs better than the classical rule-based system.

The reported results showed that our team was able to complete the challenge steps with two different methods. The final official results of our team, performed using the deep learning GoogLeNet_6 approach, achieved a challenge validation score of 0.426 and a full test score of 0.298, resulting in our team placing 12th out of 41 in the official rankings. The PhysioNet/Computing in Cardiology Challenge 2020 has provided the opportunity for unbiased and comparable research for testing the complexity of 12-lead ECG classifiers with a large public training set, as well as undisclosed validation and test sets.

Among the topics open for future investigations are the development of class-imbalance analysis, multi-label datasets and unequal sample sizes, in addition to the combination of the two proposed methods.

## Figures and Tables

**Table 1:** Composition of the considered learning/test datasets with total and reduced (/24) numbers of classes.

Database		Learning Set	Val-Test Set	Total Classes	Classes (/24)
CPSC	China Physiol. Signal Challenge 2018 [14]	6877	2926	9	6
CPSC-Extra	China 12-Lead ECG Challenge [14]	3453		72	20
INCART	St Petersburg 12-lead Arrhythmia [15]	74		37	8
PTB	PTB Diagnostic ECG [16]	516		17	4
PTB-XL	PTB-XL electrocardiography Database [17]	21,837		50	22
G12EC	Georgia 12-Lead ECG Challenge [11]	10,344	10,334	67	22
Und	Undisclosed [11]		10,000		≤24

**Table 2:** Distribution of the 24 diagnostic classes in the entire learning database and in the subset LS_N1000. * Equivalent classes.

	Code	Diagnosis	Instances (All)	LS_N1000 (Weighted)
01	IAVB	1st degree AV block	2394	1536
02	AF	Atrial Fibrillation	3475	1626
03	AFL	Atrial Flutter	314	275
04	Brady	Bradycardia	288	269
05	CRBBB *	Complete Right Bundle Branch Block	3085	737
	RBBB *	Right Bundle Branch Block		
06	IRBBB	Incomplete Right Bundle Branch Block	1611	1022
07	LAnFB	Left anterior Fascic Block	1806	972
08	LAD	Left Axis Deviation	6086	1477
09	LBBB	Left Bundle Branch Block	1041	468
10	LQRSV	Low QRS voltage	556	441
11	NSIVCB	Nonspec intrav cond disorder	997	575
12	PR	Pacing Rhythm	299	286
13	PAC *	Premature Atrial Contraction	1944	884
	SVPB *	Supraventricular prem beats		
14	PVC *	Premature Ventr Contraction	553	333
	VEB *	Ventricular ectopic beats		
15	LQT	Long QT	1513	713
16	LPR	Long PR	340	140
17	QAb	Q wave abnormal	1013	523
18	RAD	Right axis deviation	427	207
19	SA	Sinus Arrhythmia	1240	641
20	SB	Sinus Bradycardia	2359	568
21	NSR	Normal Sinus Rhythm	20,846	1000
22	STach	Sinus Tachycardia	2402	640
23	Tab	T wave abnormal	4673	455
24	TInv	T inverted	1112	214
		TOTAL	60,373	16,002

**Table 3:** Characteristics of the 3 subsets of the learning databases obtained with under-sampling.

Identification	N_Max	Number of ECG Records
LS_N600	600	11,210
LS_N1000	1000	16,002
LS_N1500	1500	20,044

**Table 4:** Definitions of the main ECG parameters, computed with classical algorithms.

**max_QRS_ampl**	Maximal amplitude of the detected QRS complexes
**min_QRS_ampl**	Minimal amplitude of the detected QRS complexes
**mean_QRS_ampl**	Mean value of all QRS amplitudes
**mean_RR**	Mean value of all RR intervals
**std_RR**	Standard deviation of of all RR intervals
**n_cross_inside_QRS**	Number of zero crossing of the first derivative inside QRS
**n_cross_outside_QRS**	Number of zero crossing of the first derivative outside QRS (illustrated in Figure 1).
**positive/negative_P_wave**	Positive/negative_P_wave in presence of a positive/negative wave in the interval [QRS_onset-400 ms, QRSonset-40 ms] with a significant peak of 20 uV within an interval of ±40 ms.
**no_Q_wave**	Absence of Q wave
**no/yes_P_wave**	Binary tests for absence/presence of a positive/negative P_wave
**QRS_dur**	Mean QRS duration
**pred_R_wave**	Presence of a predominant R wave with at least 95% of the entire QRS area in leads I or V6
**opp_ST_T**	Displacement of ST-T wave in opposition to major deflection of QRS complex in V5.
**MW_shaped_QRS**	QRS segmentation that looks like an ‘M’ or ‘W’ (illustrated in Figure 2) in any of leads V1, V2, V3 or V4
**frontal_QRS_angle**	QRS angle in the frontal plane computed as the max deviation of QRS in the frontal plane
**ratio_Q_R**	Ratio of Q_amplitude/R_amplitude in lead I
**ratio_R_S**	Maximal ratio of R_aplitude/S_amplitude in II and III
**slope_ini_QRS**	Slope of the first 30 ms of QRS complex
**T_neg**	Binary test if the amplitude of T wave is negative

**Table 5:** Cross-validation results in the CPSC learning database (6887 records), considering 9 diagnostic classes.

Method	F_1_	F_2_	G_2_	Learning Set
Rule-based RB1	0.461	0.5110	0.269	CPSC
GoogLeNet_1	0.623	0.636	0.386	CPSC
GoogLeNet_2	0.614	0.632	0.381	CPSC
GoogLeNet_3	0.618	0.634	0.390	CPSC

**Table 6:** Challenge validation phase: submissions trained in the learning set of 43,101 records and tested in the validation set of 6630 records.

Submission Name	Score	CPU-Time (Min)	#Iterations	Learning Subset
GoogLeNet_6	0.426	1664	2 (+10)	LS_N1000
GoogLeNet_8	0.420	3714	20	LS_N1000
GoogLeNet_7	0.400	2680	10	LS_N1000
GoogLeNet_9	0.422	4029	20	LS_N600
GoogLeNet_10	0.415	2875	18	LS_N1500
rule-based RB2	0.325	33	- -	- -
rule-based RB1	0.324	62	- -	- -

**Table 7:** Cross-validation (3-fold) results of the main submissions trained and tested in the learning subsets LS_N1000 or LS_N1500.

Submission Name	Score	F_1_	F_2_	G_2_	AUROC	AUPRC	Training Set
GoogLeNet _6 *	0.497	0.343	0.470	0.199	0.858	0.440	LS_N1000
GoogLeNet _8	0.499	0.382	0.50	0.222	0.867	0.459	LS_N1000
GoogLeNet _7	0.497	0.343	0.470	0.199	0.858	0.440	LS_N1000
GoogLeNet_10 *	0.480	0.335	0.466	0.194	0.864	0.438	LS_N1500
rule-based RB2	0.348	0.285	0.333	0.151	0.659	0.203	LS_N1000
rule-based RB1	0.337	0.263	0.317	0.139	0.648	0.179	LS_N1000

* With a shorter learning process.

**Table 8:** Official final results obtained using GoogLeNet_6.

Dataset	ECG Records	Official Score	AUROC	AUPRC	F_1_
Validation Set	6630	0.426	0.830	0.314	0.296
Test set—Hidden CPSC Set	1463	0.452	0.882	0.619	0.116
Test set—Hidden G12EC Set	5167	0.421	0.799	0.312	0.304
Test set—Hidden Undisclosed Set	10,000	0.205	0.810	0.376	0.244
Test Set—total	16,630	0.298	0.777	0.302	0.266

**Table 9:** Complete results for the official Challenge Validation phase: AUROC, AUPRC and F_1_ scores of the diagnostic classes for the submitted method, GoogLeNet_6, in the official test set. No results for LPR.

Diagnostic Code	AUROC	AUPRC	F_1_
IAVB	0.769	0.254	0.297
AF	0.931	0.751	0.64
AFL	0.836	0.215	0.184
Brady	0.798	0.001	0.0
CRBBB	0.913	0.642	0.51
IRBBB	0.814	0.125	0.162
LAnFB	0.862	0.143	0.172
LAD	0.821	0.257	0.325
LBBB	0.975	0.667	0.397
LQRSV	0.819	0.126	0.144
NSIVCB	0.749	0.049	0.064
PR	0.698	0.019	0.05
PAC	0.847	0.394	0.378
PVC	0.793	0.132	0.231
LQT	0.775	0.276	0.329
QAb	0.724	0.078	0.112
RAD	0.842	0.025	0.053
SA	0.93	0.47	0.425
SB	0.979	0.918	0.799
NSR	0.834	0.521	0.543
STach	0.976	0.77	0.731
Tab	0.709	0.268	0.379
TInv	0.692	0.121	0.176

## Data Availability

Publicly available datasets were analyzed in this study. These data can be found here: https://physionet.org/content/challenge-2020/1.0.1/ (accessed on 23 April 2020).
